# Does Continuous Injection Pressure Monitoring Reliably Detect Interfascial Planes in Regional Anesthesia? A Pilot Study of New Measurement System

**DOI:** 10.3390/jcm14145112

**Published:** 2025-07-18

**Authors:** Mateusz Wilk, Małgorzata Chowaniec, Karol Jędrasiak, Aleksandra Suwalska, Mariusz Gałązka, Piotr Wodarski

**Affiliations:** 1 Collegium Medicum, WSB University, 41-300 Dabrowa Gornicza, Poland; 2 Department of Anesthesiology and Intensive Care, University Clinical Center, 40-055 Katowice, Poland; 3 Department of Anatomy, Faculty of Medical Sciences in Katowice, Medical University of Silesia, 40-055 Katowice, Poland; 4 Department of Transport and Computer Science, WSB University, 41-300 Dabrowa Gornicza, Poland; kjedrasiak@wsb.edu.pl; 5 Department of Data Science and Engineering, Faculty of Automatic Control, Electronics and Computer Science, Silesian University of Technology, 44-100 Gliwice, Poland; 6 Design Department of Environmental, Meteorological and Hydrological Measurement Devices, Technika IT Sp. Z o.o. (LL Company), 44-102 Gliwice, Poland; 7 Department of Biomechatronics, Faculty of Biomedical Engineering, Silesian University of Technology, 44-100 Gliwice, Poland; piotr.wodarski@polsl.pl

**Keywords:** interfascial plane, injection pressure, regional anesthesia, needle, ultrasound

## Abstract

**Background/Objectives:** The accurate localization of interfascial planes is critical for effective regional anesthesia, yet current techniques relying on ultrasound guidance can be challenging, especially in obese or pediatric patients. Previous cadaveric and clinical studies have suggested that injection pressure varies depending on needle placement relative to fascial and neural structures. This pilot study aimed to evaluate whether the continuous monitoring of injection pressure can reliably differentiate interfascial spaces from surrounding anatomical structures in a porcine tissue model. **Methods:** A custom-built pressure monitoring system was used to continuously measure saline injection pressure during regional block procedures performed on porcine thighs. Injections were guided by ultrasound and conducted using an infusion pump. Needle positions were classified as intramuscular, resting on fascia, or interfascial. Statistical comparisons of pressure levels, variability, and temporal trends were conducted using Wilcoxon signed-rank tests and regression analysis. **Results:** Mean intramuscular pressure was significantly higher than the mean interfascial pressure (*p* < 1 × 10^−13^). Interfascial injections demonstrated lower pressure variability (*p* = 2.1 × 10^−4^) and an increasing trend in pressure over time (*p* = 2.1 × 10^−4^), whereas intramuscular injections exhibited a decreasing pressure trend (*p* = 3.15 × 10^−3^). **Conclusions:** Continuous pressure monitoring effectively distinguishes interfascial from intramuscular and fascial penetration phases during regional anesthesia. The method demonstrates potential as a real-time, objective tool for enhancing needle guidance and improving the safety and accuracy of interfascial plane blocks. Further cadaveric and clinical studies are warranted to validate these findings.

## 1. Introduction

Regional blocks are divided into plexus and peripheral nerve blocks and fascial plane blocks. The idea of fascial plane blocks is to deliver a local anesthetic into the space between the muscle fascias, where there are nerves covering significant areas of the human body [1]. The fascial plane block technique involves locating this very space with the tip of the needle, which is not always easy, especially in obese patients, patients with chronic inflammatory connective tissue diseases, and patients suffering from chronic pain [2,3]. In these groups of patients, the fascia is often a tough tissue, difficult to penetrate with a needle, resulting in the risk of puncturing through the fascial space and administering the local anesthetic intramuscularly, which will result in ineffective blockade and myotoxicity generated by the LMZ [4]. The opposite situation is observed in the pediatric population, in whom the fascias are perfectly visible using ultrasound; however, they are considerably more elastic, which also makes it difficult to precisely locate the needle tip in the fascial space [5]. Technical difficulties due to the thickness of the tissues and their translucency for ultrasound also occur in obese patients, in whom the fascial planes may be poorly visible and thus more difficult to locate with the needle tip [6]. In previous years, beginning from 2014 [7,8,9,10], studies suggesting that opening injection pressures >15 PSI (pound per square inch) indicated intraneural injection of local anesthetic. In 2016, a study by Gadsen J [11] showed that needle tip contact with a fascia produced opening injection pressure >15 PSI. The first system for the continuous measurement of local anesthetic injection pressure was proposed in 2018 by Quadri C [12] and was later used in continuation studies [13,14,15,16]. This system, however, used optic fiber, and only small volumes of either saline or local anesthetic were used. In our study, we propose a simpler measurement system based on a pressure detector with continuous injection speed and, thus, continuous pressure monitoring. We also propose the continuation of infusion during the whole procedure, contrary to previous studies where injection pressure was measured after the proper localization of the needle tip.

### Purpose of This Study

The purpose of this study was to answer the following research questions:

1. Does the value of intramuscular injection pressure differ from interfascial injection pressure?
2. Does the value of injection pressure during the passage through the fascia differ from intramuscular and interfascial injection pressures?
3. Is the value of interfascial injection pressure constant or variable, and if variable, what is the trend?

## 2. Materials and Methods

Before beginning the studies, approval from the Local Ethical Committee for Animal Experiments of Medical University of Silesia in Katowice, Poland, was obtained (issue BNW/NWN/0052/LKE/4/24).

**Figure 1:**
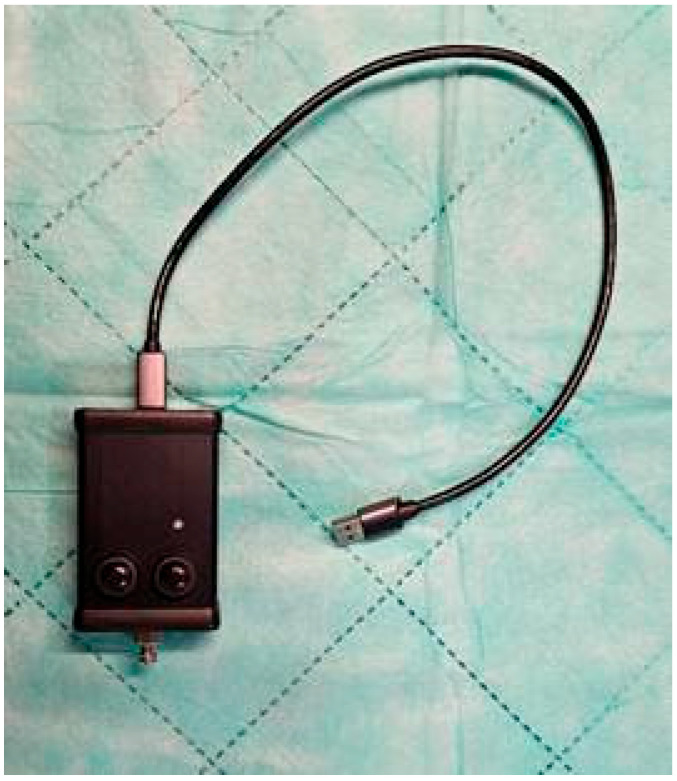
Injection pressure measuring device.

Prior to the research, based on grant funds RW/36/25, a device for measuring the pressure of liquids was built. The device (Figure 1) consisted of a pressure sensor, a docking port, a button to start recording, a button to mark “events” during recording, and LEDs to indicate operation. The device was powered by the USB port of the computer to which it was connected. The same USB port was used to send data to the computer. A program compatible with the device to record measurement data was written. The device showed an accuracy of ±0.25% (±0.145 PSI), a pressure measurement range of 0–58 psi (0–4 bar), an operating temperature range of −40–120 °C, and a 24-bit analog-to-digital converter. The energy requirement was less than 0.1 mW for a sampling frequency of 10 Hz. The measuring membrane used in the device was coated with gel to protect against aggressive media. A typical sensor supply voltage of 3.3 VDC was used. There was a Luer Lock connector on the housing of the device. The connector, through a flexible tube, brings the test medium to the measuring cell. An analog-to-digital converter integrated in the pressure sensor measures changes in the stress of the measuring cell, which are directly proportional to the measured pressure. The measured values are read out by a microcontroller via a serial interface and then converted.

The measurement values calculated in this way are sent to the computer via the USB interface at the appropriate frequency. Along with the measurements, button information also reaches the application.

**Figure 2:**
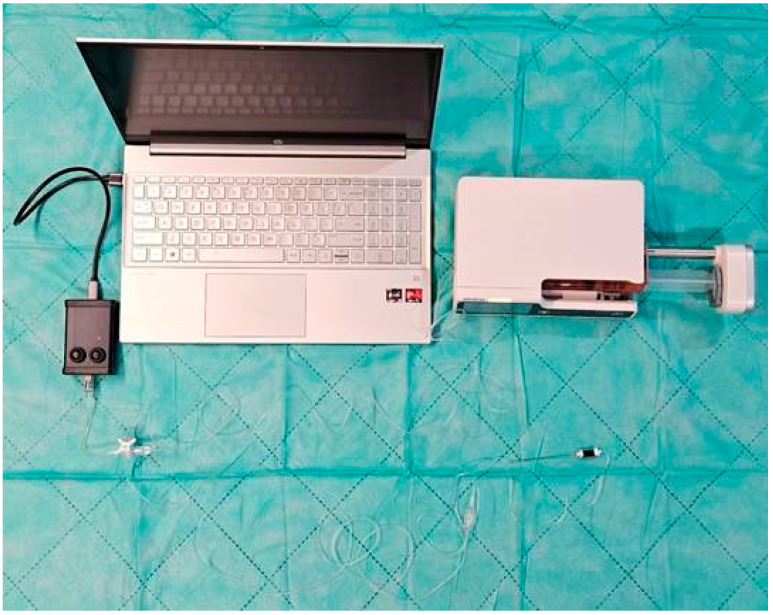
Measurement system.

The measuring system (Figure 2) consisted of a Mindray BeneFusion infusion pump, a 60 mL BD PlasticPack infusion syringe, 2 × 150 cm drains, a three-way stopcock, a measuring device, and a regional block needle.

The animal material used in this study was 4 porcine thighs. SonoBlock II Facet 22G × 80 needles provided by Pajunk were used in this study. A Mindray TE 7 ultrasound machine with a linear probe was used for tissue imaging.

**Figure 3:**
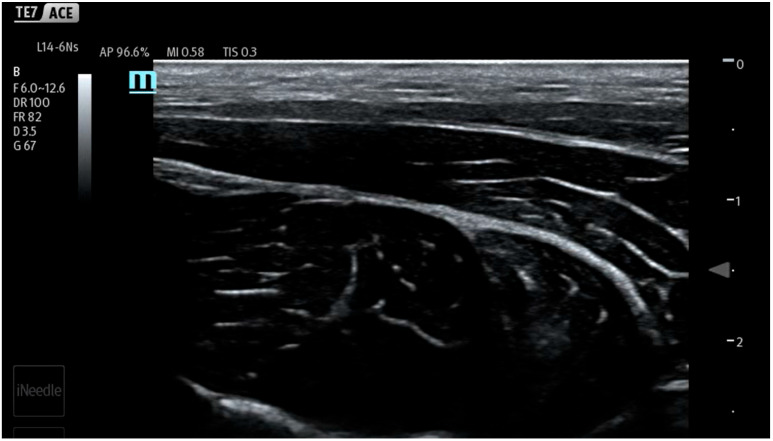
Ultrasound view of porcine thigh structures consisting of skin, muscles, and fascia.

**Figure 4:**
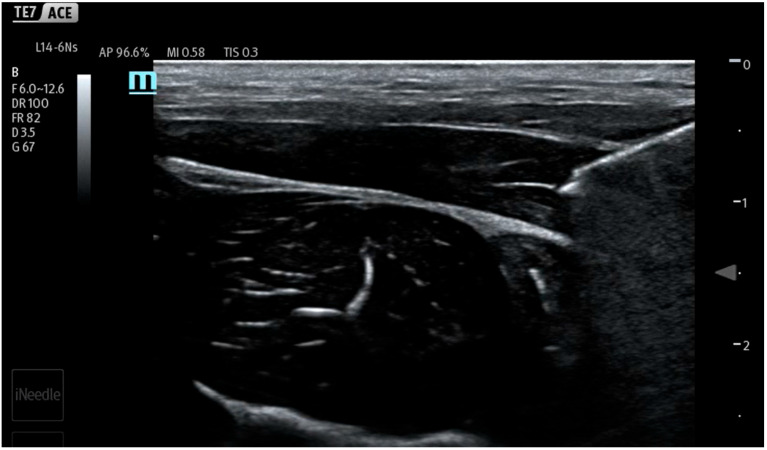
Needle tip in intramuscular position.

**Figure 5:**
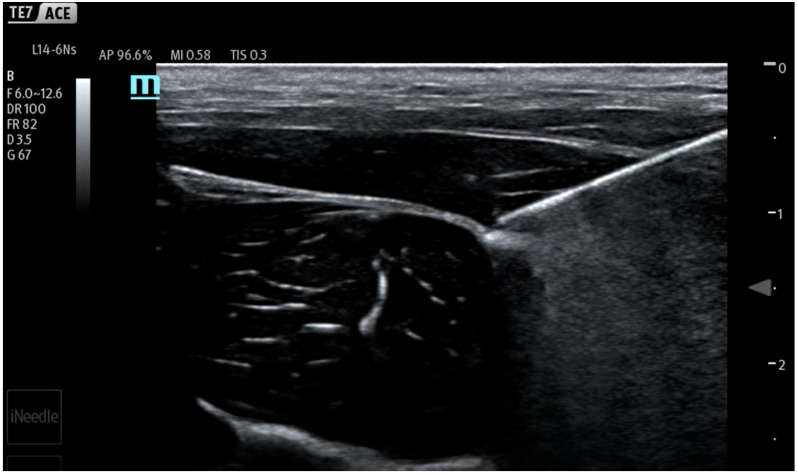
Needle tip resting on fascia.

During the study, saline was continuously administered using an infusion pump at a rate of 10 mL/min (600 mL/h) to conform to the methodology of previous works [7,8,9,10,11,12]. Different from previous studies, the infusion and measurements were started before injection, continued during needle maneuvers in the tissues and the hydrodissection of the interfascial plane, and ended after needle removal from the tissues. In the first stage of the study, the fluid pressure generated by the measuring system (a needle not inserted into the tissue) was checked (Figure 3). It turned out that the system generates a plateau pressure of 2.3 PSI. After the pressure graph stabilized (plateau phase), a needle was inserted in tissues aiming towards the interfascial plane (Figure 4). During the whole procedure, saline was continuously administered through an infusion pump, which enabled pressure monitoring during needle propagation through subsequent tissue layers (Figure 5).

**Figure 6:**
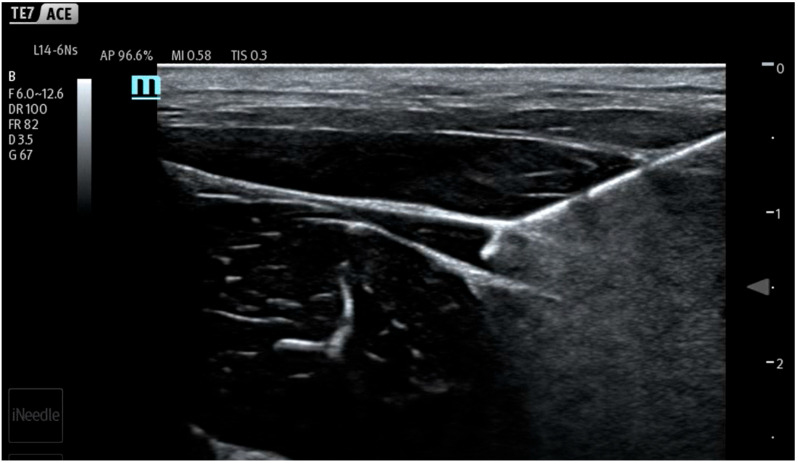
Needle tip inside interfascial space with visible hydrodissection.

**Figure 7:**
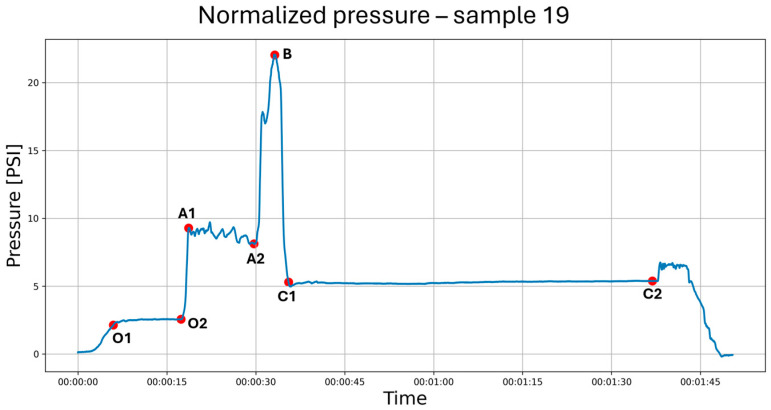
Exemplary pressure curve recording with extreme points marked. Section O1–O2—Pressure plateau phase resulting from the resistance of the measuring system. Tissue injection started from point O2. A1–A2—Intramuscular needle movement. B—Resting of the needle tip on the fascia. C1–C2—Needle tip in the interfascial space, hydrodissection. PSI—pound per square inch.

After locating the interfascial plane (Figure 6) with the needle tip, a 60 s time was timed to measure the interfascial injection pressure (Figure 7). In the course of the study, 10 mL of saline was introduced into each interfascial space. Five separated interfascial hydrodissections were performed in different interfascial planes in every thigh, respectively. In total, 20 separated hydrodissections were performed.

In this study, an anesthesiologist performed the in-plane puncture of the tissues of the porcine thigh, directing the needle into randomly selected interfascial spaces until a hydrodissection of the fascias of adjacent muscles was achieved (kayak sign). The operator saw only the image on the ultrasound machine, and the results of the measurements were blinded for him. During the measurements, the moments of passing through the muscle, resting on the fascia, puncturing the fascia, and the hydrodissection of the interfascial space, as well as the exit of the needle from the fascial space, were noted in the measurement program. The data collected in this way were exported to Excel files. Statistical analysis was performed.

### Statistical Analysis

All injection pressure data were initially normalized by subtracting the baseline pressure, calculated as the mean pressure recorded prior to needle insertion (segment O). These normalized values were used for all subsequent analyses to ensure standardized comparisons across procedures. Mean injection pressures were calculated for the intramuscular and interfascial phases (segments A and C, respectively), while the pressure at fascial puncture (point B) was extracted as a single peak value. Pressure variability was assessed by calculating the standard deviation within segments A and C. Prior to conducting statistical tests, the normality of the distributions was evaluated using the Shapiro–Wilk test. In cases where normality was not confirmed, non-parametric tests were conducted.

Differences in mean pressures across all injection phases (intramuscular, interfascial, and fascial puncture) were assessed using the Friedman test for repeated measures. Where global differences were identified, post hoc or pairwise Wilcoxon signed-rank tests were conducted. Multiple comparisons were corrected using the Benjamini–Hochberg false discovery rate method. Effect sizes (point-biserial rank correlation) were calculated for all pairwise comparisons to quantify the magnitude of observed differences. Comparisons of pressure variability between intramuscular and interfascial phases were performed using the Wilcoxon signed-rank test.

To assess directional trends in pressure changes during injection, linear regression analyses were conducted separately for each measurement series within the intramuscular and interfascial phases. Regression slopes were extracted and subsequently analyzed using the Wilcoxon signed-rank test to determine whether systematic increasing or decreasing trends were present. All statistical analyses were performed using a statistical significance level of 0.05.

## 3. Results

### 3.1. Comparison of Mean Pressure Values

To evaluate differences in mean injection pressures between the intramuscular and interfascial spaces, the Shapiro–Wilk test was applied to assess the normality of paired differences. The analysis indicated a significant deviation from normality (*p* = 3.301 × 10^−3^).

**Figure 8:**
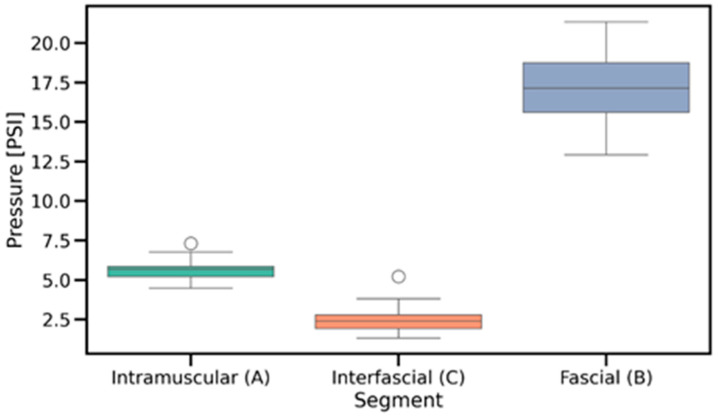
Mean injection pressures across intramuscular, interfascial, and fascial puncture locations. PSI—pound per square inch.

**Figure 9:**
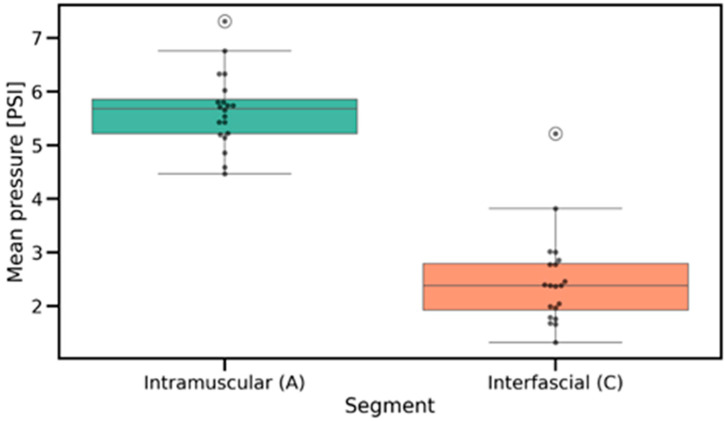
Distribution of mean injection pressures in intramuscular and interfascial spaces, highlighting differences in pressure levels and within-group variability. PSI—pound per square inch.

**Figure 10:**
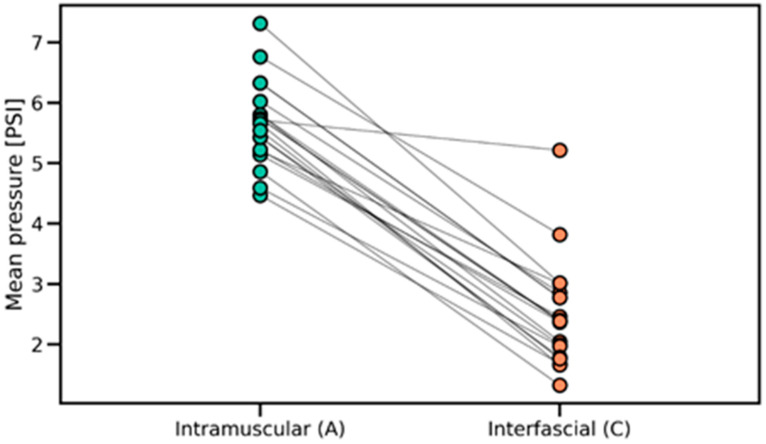
Paired comparisons of mean injection pressures between intramuscular and interfascial spaces, showing individual transitions across injection phases. PSI—pound per square inch.

The Friedman test assessed the differences in pressure values recorded during fascial penetration, relative to intramuscular and interfascial injections (Figure 8), which revealed significant overall differences across the examined anatomical locations (*p* = 2.061 × 10^−9^). Post hoc Wilcoxon signed-rank tests with Benjamini–Hochberg correction confirmed that pressure at the fascial puncture was significantly higher compared to both interfascial (*p* = 5.192 × 10^−17^) and intramuscular injection pressures (*p* = 7.136 × 10^−16^). Additionally, intramuscular pressure was significantly greater than interfascial pressure (*p* = 3.520 × 10^−13^), as shown in Figure 9 and Figure 10. The provided effect sizes indicate a very strong effect (>0.8). These results, presented in Table 1, indicate a systematic and pronounced pressure increase during fascial puncture compared to the more stable profiles observed during intramuscular and interfascial injection phases.

### 3.2. Comparison of Pressure Variability

**Figure 11:**
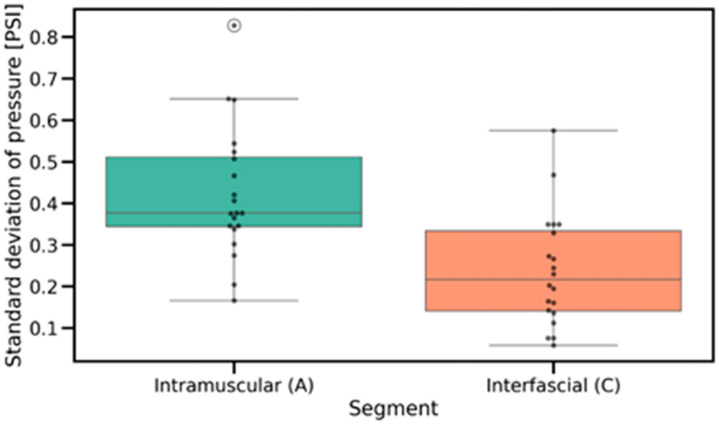
Distribution of pressure variability in intramuscular and interfascial spaces, illustrating differences in within-phase pressure stability. PSI—pound per square inch.

**Figure 12:**
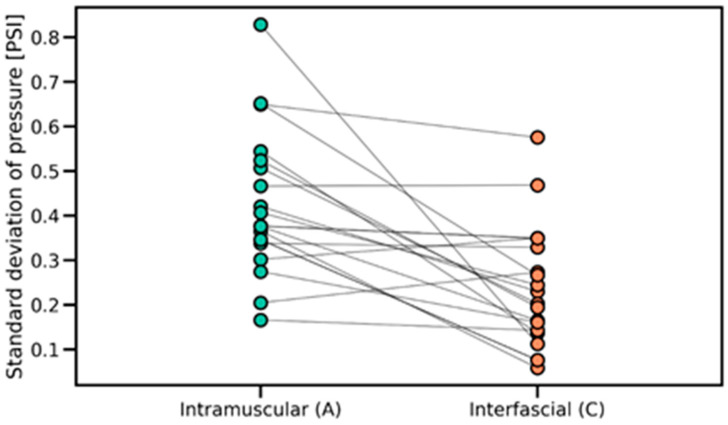
Paired comparisons of pressure variability between intramuscular and interfascial spaces, showing individual transitions and confirming reduced variability during interfascial injection. PSI—pound per square inch.

The Wilcoxon signed-rank test was applied to compare pressure variability between the intramuscular and interfascial spaces (Figure 11 and Figure 12). The analysis revealed a significant difference (*p* = 2.100 × 10^−4^), indicating lower pressure variability during interfascial injection (Table 1). This suggests a more stable pressure profile in the interfascial space compared to the intramuscular space. Table 2 includes the pressure characteristics computed across all injection measurements, which were used for statistical testing.

### 3.3. Trend Analysis

To assess the presence of directional trends in pressure changes over time, linear regression analysis was performed for each measurement series separately within the intramuscular and interfascial spaces. For each case, the regression slope relative to normalized time was calculated, and the Wilcoxon signed-rank test was applied to test the null hypothesis of no trend (median slope = 0).

**Figure 13:**
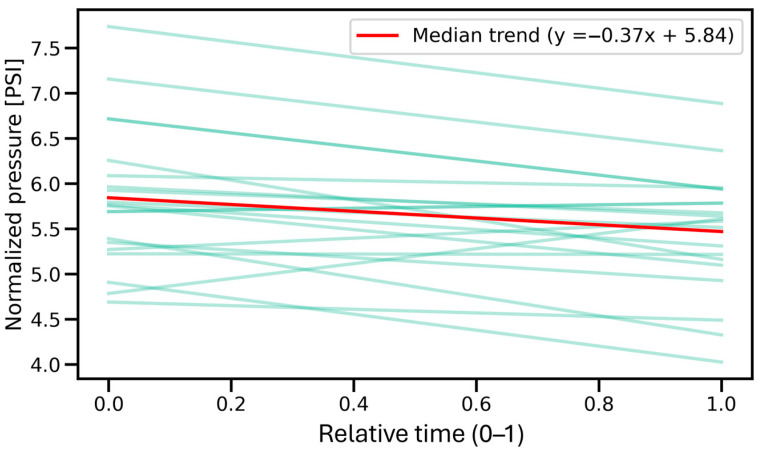
Regression lines (green) illustrating pressure trends during intramuscular injection across all measurements. The median regression line (in red) reflects a significant decreasing trend, indicating a systematic decline in pressure during this phase. PSI—pound per square inch.

**Figure 14:**
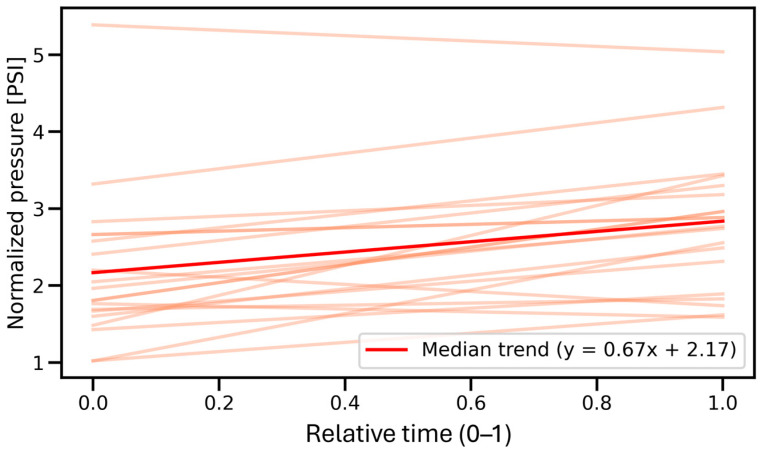
Regression lines (orange) illustrating pressure trends during interfascial injection across all measurements. The median regression line (in red) reflects a significant increasing trend, indicating a gradual rise in pressure over time during this phase. PSI—pound per square inch.

In the intramuscular space, as shown in Figure 13, the median slope was –0.3737, and the Wilcoxon test revealed a significant decreasing trend (*p* = 3.153 × 10^−3^), indicating that injection pressure tended to decline during the procedure. In contrast, the interfascial space (Figure 14) demonstrated a significant increasing trend, with a median slope of 0.6696 (*p* = 2.100 × 10^−4^), reflecting a gradual rise in pressure over time. These findings confirm distinct temporal dynamics of injection pressure within the two anatomical spaces.

## 4. Discussion

It should be noted that there are few studies strictly describing the hydrodissection pressures of interfascial spaces. In our method, we wanted to rely on continuous measurement of the infusion pressure at a constant (set at 10 mL/min based on the literature [7,8,9,10,11,12]) flow rate. After the injection pressure stabilized at the plateau phase, which was 2.3 PSI for our measurement system, we inserted the needle into the tissue by measuring the pressure accompanying the infusion. Given the data analysis methodology, it can be assumed that the results obtained during the study take into account the pressures at the tip of the needle (the data obtained during the experiments were related to the plateau pressure described above as the O2 point, which in the data analysis was pressure = 0).

In 2020, a paper by Capdevila M et al. [14] was published, which described continuous saline administration while performing a regional block with simultaneous measurement of injection pressure of the agent in the muscle, perineuraly, intraneurally, and resting on the fascia, while performing sciatic, femoral, and median nerve blocks on the arm and forearm. Saline was administered at different rates, 1.2, 5, and 10 mL/h. For each measurement, no more than 1.5 mL of solution was administered. Statistically significant differences were shown between the different needle locations.

Also, in 2020, a paper by Saporito A [15], in which brachial plexus blocks were performed supraclavicularly in two cadavers, with a total of 15 perineural, intraneural, and intravenous injections. The study showed significant differences in the injection pressure of 1 mL saline to each location, with intravenous injection having the lowest injection pressure. As only a very small volume was injected, it is difficult to compare these papers with our research.

As we mentioned in the Introduction, a paper by Quadri C et al. [12] was published in 2018 describing a novel method for the fiber-optic measurement of needle tip pressure thanks to an optical fiber inserted through the needle shaft into the needle tip. In our system, we used the plateau pressure (O2) to determine relevant pressure = 0, and each pressure change was the same as the pressure at the needle tip. Compared to this work, we did not inject the saline intraneurally, but rather interfascially; hence, our average pressure result (2.48 PSI, 10 mL volume) was lower than that obtained by the Quadri team in the needle tip measurement (27.1 kPa = 3.93 PSI, 2 mL volume perineurally). Also, in 2018, a paper was published by the team of Saporito A et al. [13], who performed injections simulating a sciatic nerve block. Each injection was performed at the corresponding level of the same sciatic nerve with the sensing needle placed in both the perineural space and intraneurally. The following locations were chosen: gluteal; sub-gluteal; mid-femoral; and popliteal. At each site, a total of 5 mL saline was injected at a constant rate (10 mL·min^−1^) via an electronic pump.

Another study by Gadsen J [11], conducted on a group of 20 patients, showed that the opening injection pressure was >15 PSI (pound per square inch) for 90% of cases of the needle tip adherence to the femoral nerve and 100% of cases of the needle tip adherence to the fascia iliaca. The study was performed at four locations: indenting the fascia iliaca, advanced through the fascia iliaca while lateral to the nerve, slightly indenting the femoral nerve, and withdrawn from the nerve by 1 mm. At each location, the OIP required to initiate an injection of 1 mL D5W (5% dextrose in water) at 10 mL/min (600 mL/h) was recorded. It was found that the pressure of the perineural injection (and therefore de facto injection to the interfascial space containing the nerve) ranged from 3.8 to 6.1 PSI, which, given that in our study we did not examine the plateau pressure resulting from the resistance of the test system, allows us to conclude that in the case of the interfascial hydrodissection pressure we obtained, the results were very similar (our mean pressure of 2.48 PSI + plateau pressure of 2.3 PSI = 4.78 PSI). Compared to the study by Krol et al. [9], we obtained significantly lower hydrodissection pressures compared to those outlined above; however, again, these were cadaveric studies and involved nerve and plexus blocks. Compared to the work of Roberto D et al. [16], which indicated that the continuous monitoring of infusion pressure can help localize the needle tip in the transversus abdominis plane in human cadaver studies, we obtained lower interfascial space hydrodissection pressures (Roberto D 30.99 kPa (4.49 PSI) vs. our study (2.48 PSI)), but once again, our pilot study was based on the interfascial injection pressure of porcine thigh, not human cadaver TAP. In comparison to the study by Steinfeldt T et al. [17], which used a needle tip pressure monitoring system analogous to that proposed by Quadri, conducting the study on cadavers yielded an average perineural injection pressure measured at the needle tip of 2.3 PSI (very close to the 2.48 PSI obtained by our team). The above considerations indicate the need for further research in this field.

An important finding is the fact that an upward trend in pressure was detected in the fascial space (Figure 14), indicating a slow distribution of saline in the space, and little variation in pressure during the injection (Figure 10), indicating a stable position of the needle tip in the interfascial space and a fairly loose connection of the fascias between each other.

The pressure measured as it passed through the muscle (Figure 13) showed considerable variability (Figure 11) due to the muscle fibers encountered and punctured by the needle. An interesting finding is the downward trend in pressure as the needle passes through the muscle, which is most likely related to its forward motion during puncture. Similarly to other studies [11,16], the moment the needle rested on the fascia (Figure 8, point B in Figure 7) caused a significant increase in pressure, averaging to more than 17 PSI.

The measurement method proposed by our team makes it possible to accurately determine the fascial space. As interfascial spaces are often difficult to hydrodissect, the usage of continuous infusion speed and pressure monitoring enables easier detection of interfascial plane needle tip positioning. If saline solution as a safety measure rather than local anesthetic is used to find it, enhancing needle guidance and control during block performance, and then local anesthetic solution is injected to hydrodissected interfascial space, it makes it possible to increase patient safety by minimizing the myotoxicity of local anesthetic drugs, increasing the success rate of interfascial plane blocks and ultimately improving the safety and efficacy of the block itself.

## 5. Conclusions

Based on the results, it can be concluded that the continuous measurement of the injection pressure of saline during the passage of the regional block needle through the tissues differs significantly between intramuscular injection and the contact of the needle tip with the fascia and interfascial space injection. Simultaneously, this method makes it possible to accurately determine between the moment of needle–fascia contact and entry into the interfascial space. The results indicating the variation and upward trend of saline injection pressure in the fascial space encourage cadaveric studies.

## Figures and Tables

**Table 1:** Summary of statistical comparisons for mean injection pressures and pressure variability between intramuscular, interfascial, and fascial puncture phases.

	Test	Comparison	StatS	*p*-Value	adj. *p*-Value	Effect Size
MEAN	Friedman	A vs. C vs. B	40.00	2.0612 × 10^−9^	-	-
	Wilcoxon	B vs. A	30.07	1.7356 × 10^−17^	5.1917 × 10^−17^	0.9641
	Wilcoxon	B vs. C	25.15	4.7571 × 10^−16^	7.1356 × 10^−16^	0.9762
	Wilcoxon	C vs. A	−17.51	3.5194 × 10^−13^	3.5194 × 10^−13^	−0.9001
STD	Wilcoxon	A vs. C	14.00	2.1000 × 10^−4^	-	0.8667

**Table 2:** Summary of calculated pressure characteristics across all injection measurements. For each case, the mean minimum, maximum, and standard deviations of injection pressures were determined for the intramuscular (segment A) and interfascial (segment C) phases. In addition, the peak pressure recorded at fascial puncture (point B) was extracted.

	Intramuscular				Interfascial				Fascial
Sample	Mean	Min	Max	Std	Mean	Min	Max	Std	Pressure
1	5.20	4.14	7.60	0.54	3.01	2.34	3.20	0.14	16.40
2	4.47	3.74	5.47	0.37	1.68	1.57	1.82	0.06	16.40
3	4.59	4.05	5.00	0.17	1.97	1.76	2.29	0.14	12.92
4	5.71	4.69	10.91	0.83	5.21	5.04	5.50	0.11	18.41
5	6.33	5.60	7.21	0.35	2.77	2.47	2.90	0.08	19.54
6	5.65	4.61	6.30	0.38	1.66	1.45	2.01	0.16	14.43
7	5.43	4.33	6.65	0.47	1.79	1.10	2.37	0.47	17.85
8	4.86	3.69	7.38	0.52	1.32	1.08	1.72	0.19	20.12
9	5.54	4.85	6.68	0.27	1.76	1.50	2.18	0.16	15.81
10	6.76	5.90	7.87	0.34	3.82	3.41	4.44	0.33	15.50
11	5.80	4.96	6.65	0.30	2.05	1.56	2.66	0.35	17.11
12	5.74	4.65	6.73	0.38	2.39	1.90	2.87	0.35	15.60
13	5.43	4.40	6.72	0.42	1.99	1.67	2.61	0.23	14.24
14	5.14	3.14	8.56	0.65	2.46	1.64	3.32	0.58	19.37
15	6.02	5.53	6.52	0.20	2.85	2.41	3.25	0.27	17.15
16	5.80	4.72	8.37	0.51	2.40	2.04	2.75	0.20	18.54
17	5.22	4.18	6.26	0.41	2.37	1.90	2.87	0.24	18.50
18	7.31	5.29	11.19	0.65	3.01	2.62	3.47	0.27	21.33
19	6.33	5.60	7.21	0.35	2.77	2.47	2.90	0.08	19.54
20	5.74	4.65	6.73	0.38	2.39	1.90	2.87	0.35	15.60
Mean	5.65	4.64	7.30	0.42	2.48	2.09	2.90	0.24	17.22
Std	0.69	0.72	1.54	0.16	0.87	0.89	0.88	0.14	2.21

## Data Availability

Data are attached as Supplementary Material in a separate file.

## Supplementary Materials

The following supporting information can be downloaded at https://www.mdpi.com/article/10.3390/jcm14145112/s1.
